# 2250. Fluoroquinolone Stewardship at a Veterans Affairs Facility: A Six Year Review

**DOI:** 10.1093/ofid/ofad500.1872

**Published:** 2023-11-27

**Authors:** Jessica Glicksberg, Morgan Froehlich, Mary Creed, George Psevdos

**Affiliations:** Stony Brook Medical Center, Stony Brook, New York; Northport VAMC, Northport, New York; Northport VA, Northport, NY; Northport VA Medical Center, Northport, New York

## Abstract

**Background:**

Increased utilization of antibiotics leads to decreased antimicrobial susceptibility, a well-established phenomenon. Antimicrobial stewardship programs (ASP) promote appropriate prescribing of antibiotics by decreasing inappropriate use, aiming to improve patient outcomes and reducing antimicrobial resistance. Fluroquinolones (FQ) are among the most prescribed antibiotics. We describe out institutional FQ use since inception of our ASP and followed the trend for susceptibility of Enterobacteriaceae and Pseudomonas

**Methods:**

Northport Veterans Affairs Medical Center (VAMC) provides care for over 30000 Veterans in Long Island, New York, with emergency room, acute care hospital, nursing homes and outpatient clinics A Retrospective review investigated FQ use by total refills per year and by days of therapy per 1000 patient days at Northport VAMC from 2012 to 2022, along with review of local annual antibiograms focusing on ciprofloxacin susceptibility to *E. coli, K. pneumoniae, P. mirabilis* and *P. aeruginosa.* An ASP in our facility was initiated in 2016. FQs are restricted antibiotics and require approval by the infectious diseases team (attendings, fellows, pharmacist)

**Results:**

Figure 1 provides the outpatient total fills per year for Ciprofloxacin, Levofloxacin, Moxifloxacin. Inpatient FQ use during the ASP years was low: Figure 2 shows the FQ inpatient use (days of therapy/1000days) from 2019 to 2022. Comparing years 2012-2016 vs 2017-2022 there was a decrease in fills of ciprofloxacin (median 604 vs. 308 fills, P: 0.009), levofloxacin (276 vs. 116, P: 0.032), and moxifloxacin (116 vs. 14, P: 0.015). By same period comparison (see figure 3), Ciprofloxacin susceptibility for *E. coli* ranged median 66.5% to 61.5%, P: 0.126, *K. pneumoniae* 67% vs. 75.5% P: 0.086, *P. mirabilis* 45% vs. 47.5% P: 0.451, and *P. aeruginosa* 63% vs. 68.5% P: 0.308

Figure 2

**Figure:**
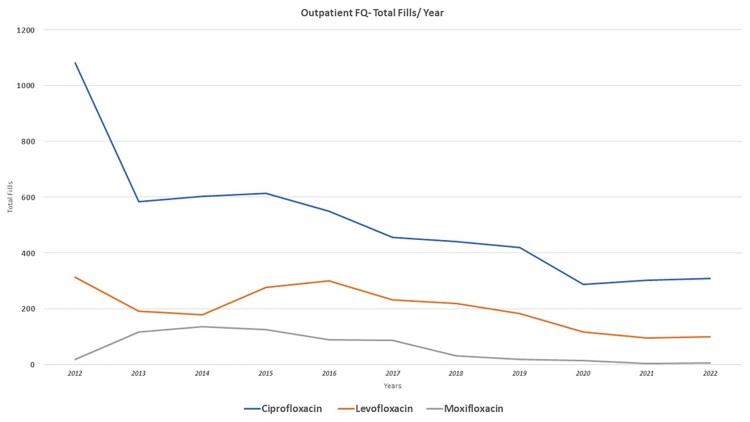

inpatient FQ use 2019 to 2022

Figure 3

**Figure:**
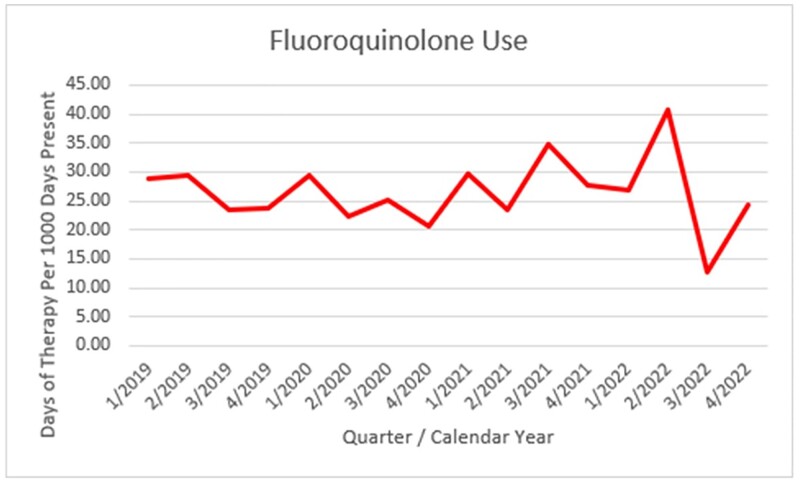

Ciprofloxacin susceptibility trend over time

**Figure:**
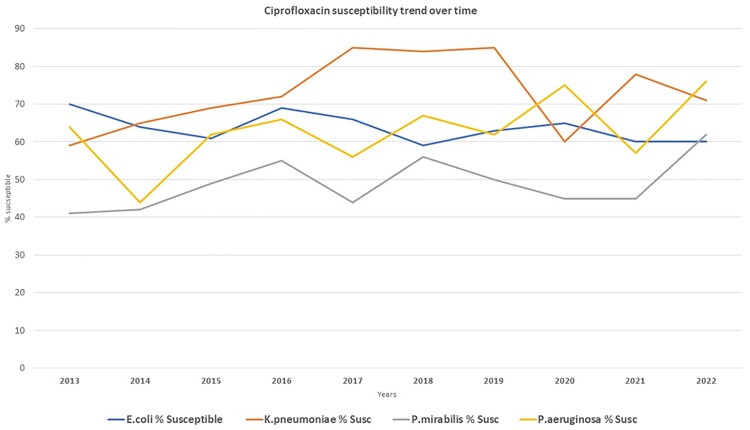

**Conclusion:**

There was a statistically significant decrease in the use of FQ in the outpatient setting after ASP was initiated in our facility. Although there was a trend in improvement in ciprofloxacin susceptibilities for Klebsiella, Proteus, and Pseudomonas, it was not statistically significant. Nevertheless, our results show that reducing FQ consumption can prevent worsening susceptibility trends for enteric gram negatives and Pseudomonas.

**Disclosures:**

**All Authors**: No reported disclosures

